# Pioneer midbrain longitudinal axons navigate using a balance of Netrin attraction and Slit repulsion

**DOI:** 10.1186/1749-8104-9-17

**Published:** 2014-07-24

**Authors:** Minkyung Kim, W Todd Farmer, Brielle Bjorke, Samuel A McMahon, Pierre J Fabre, Frédéric Charron, Grant S Mastick

**Affiliations:** 1 Department of Biology, University of Nevada, 1664 N Virginia St, Reno, NV 89557, USA; 2 Molecular Biology of Neural Development, Institut de Recherches Cliniques de Montréal (IRCM), 110 Pine Avenue West, Montreal, QC H2W 1R7, Canada

**Keywords:** Axon guidance, Floor plate, Longitudinal axon, Netrin, DCC, Robo, Slit, Hindbrain

## Abstract

**Background:**

Longitudinal axons grow parallel to the embryonic midline to connect distant regions of the central nervous system. Previous studies suggested that repulsive midline signals guide pioneer longitudinal axons by blocking their entry into the floor plate; however, the role of midline attractants, and whether attractant signals may cooperate with repulsive signals, remains unclear. In this study we investigated the navigation of a set of pioneer longitudinal axons, the medial longitudinal fasciculus, in mouse embryos mutant for the Netrin/Deleted in Colorectal Cancer (DCC) attractants, and for Slit repellents, as well as the responses of explanted longitudinal axons in vitro.

**Results:**

In mutants for Netrin1 chemoattractant or DCC receptor signaling, longitudinal axons shifted away from the ventral midline, suggesting that Netrin1/DCC signals act attractively to pull axons ventrally. Analysis of mutants in the three Slit genes, including Slit1/2/3 triple mutants, suggest that concurrent repulsive Slit/Robo signals push pioneer axons away from the ventral midline. Combinations of mutations between the Netrin and Slit guidance systems provided genetic evidence that the attractive and repulsive signals balance against each other. This balance is demonstrated in vitro using explant culture, finding that the cues can act directly on longitudinal axons. The explants also reveal an unexpected synergy of Netrin1 and Slit2 that promotes outgrowth.

**Conclusions:**

These results support a mechanism in which longitudinal trajectories are positioned by a push-pull balance between opposing Netrin and Slit signals. Our evidence suggests that longitudinal axons respond directly and simultaneously to both attractants and repellents, and that the combined signals constrain axons to grow longitudinally.

## Background

Longitudinal axons connect regions of the central nervous system by forming tracts that project long distances by growing precisely along particular trajectories. In the vertebrate embryonic brain stem, longitudinal axons are the first neurons to initiate growth. These axons originate in the midbrain and forebrain, and descend longitudinally through the brainstem to pioneer a simple scaffold of tracts [1-6]. Longitudinal trajectories require a source of cues sustained along their ipsilateral trajectories. Because they project parallel to the longitudinal axis of the brain stem, a potential source of cues for the pioneer longitudinal tracts is the floor plate tissue along the ventral midline, which produces long range and local cues [7].

Floor plate guidance cues have primarily been studied for commissural axons, which grow toward and across the midline using the floor plate as an intermediate target [8,9]. The major secreted cues for vertebrate commissural axons are Netrin1 via its main attractive receptor Deleted in Colorectal Cancer (DCC) [10-13], and the three Slits via their family of repulsive Robo receptors, primarily Robo1 and 2 [14-16]. Longitudinal axons grow parallel to the floor plate but through the same environment as commissural axons, and could be guided by the same attractive and repulsive signals. For repulsion, longitudinal axons respond to the loss of Slit/Robo signals by shifting into the ventral midline [17-20], or show altered fasciculation in other systems [19], and, furthermore, ventral pioneer axons grow around to avoid ectopic patches of Slit-expressing tissue [21,22]. In *Drosophila*, longitudinal axons express different levels or combinations of three Robo isoforms, a Robo code that sets the position of longitudinal axons from the midline [23-25]. A parallel Robo code in mice has been proposed to guide post-crossing spinal cord commissural axons [16,26]. However, Robo1 and 2 appear to guide pioneer longitudinal axons in a redundant, rather than code-like, manner [18,27].

The shift of longitudinal axons into the midline in Slit/Robo mutants implies a strong midline attractive signal. For this attraction, the most likely candidate is Netrin1. Netrin mutations in fly and worm disrupt longitudinal tracts [28-30]. However, Netrin loss in flies causes relatively mild longitudinal errors, and combined mutants suggest that Netrin is not the sole midline attractant accounting for shifts into the midline in Robo mutants [23,31,32]. Other midline attractants for vertebrate pre-crossing commissural axons collaborate with Netrin1 [33,34]. However, the role of midline attractants for longitudinal trajectories remains less defined, as are their potential interactions with Slit/Robo repellents. In zebrafish embryos, Robo2/astray mutations cause shifts of a specific population of dopaminergic longitudinal axons into and across the midline, and this midline attraction is Netrin1-dependent, as it is partially suppressed by morpholino knockdown of either Netrin1 or DCC [20]. However, Netrin1 or DCC knockdowns had no effect in a wild-type background, leaving unresolved whether Netrin/DCC signals have an independent attractive role or are instead only active when Slit/Robo signals are absent [20]. This second mechanism, implying Slit/Robo gating of Netrin1/DCC signaling, is consistent with the predominant silencing model, based on cultured *Xenopus* commissural axons which use a sequential switch from pre-crossing Netrin1/DCC attraction to post-crossing Slit/Robo repulsion [35]. This switch depends on Robo silencing DCC through binding of their intracellular domains [35]. However, in vivo genetic evidence in commissural and other axonal systems show independent action of Netrin and Slit cues [31,36] or even Netrin1 dominance that suppresses or changes Slit responses [37,38], suggesting that Netrin/Slit crosstalk can be diverse.

In this study, we set out to uncover the mechanisms by which longitudinal axons maintain specific trajectories along the dorsal-ventral (DV) axis in embryonic mice. We focused on the medial longitudinal fasciculus (MLF), pioneer axons that are the first to descend through the hindbrain adjacent and parallel to the floor plate. MLF axons are therefore exposed to high levels of Netrin and Slit signals. We tested the roles of Netrin1 and receptor signaling, and their potential interactions with Slit/Robo signaling, by assaying MLF trajectories in mutant mice, and examining direct in vitro influences on cultured axons. Together, our evidence suggests that Netrin1 attraction and Slit repulsion are balanced against each other to guide and promote the growth of longitudinal pioneer axons along a precise pathway.

## Results

### Netrin1 is expressed along the path of the medial longitudinal fasciculus

**Figure 1:**
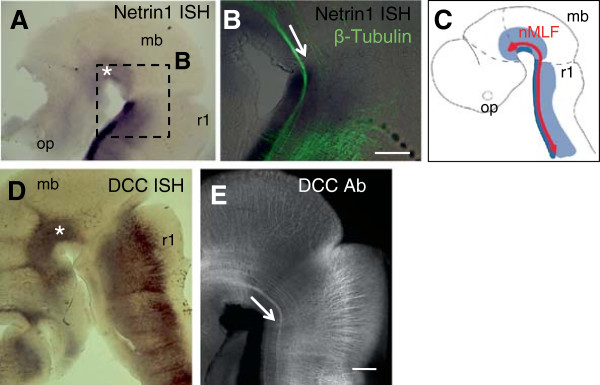
**Netrin1 and deleted in colorectal cancer receptor expression is closely associated with the medial longitudinal fasciculus in the early brain. (A-C)** Netrin1 expression pattern revealed by in situ hybridization (ISH) in embryonic day 10.5 mouse embryos. Side view of bisected neural tubes. **(A)** The location of the nucleus of the medial longitudinal fasciculus (nMLF) is indicated (*). **(B)** Close up showing axons, as labeled by anti-β-tubulin, with respect to Netrin1 expression. The medial longitudinal fasciculus (MLF) travels through the Netrin1-positive region (arrow). **(C)** Cartoon showing MLF (red) and Netrin1 expression. Dark blue is strong Netrin1 expression while light blue is weaker Netrin1 expression. **(D,E)** Deleted in colorectal cancer (DCC) expression pattern, revealed by ISH for mRNA **(D)** and DCC antibody (Ab) labeling **(E)**. The position of the nMLF is noted (*) in **(D)**, and the MLF bundle (arrow) in **(E)**. Scale bar in **(B)** indicates 200 μm; scale bar in **(E)**, 200 μm, applies to **(A,D,E)**. mb, midbrain; op, optic vesicle; r1, rhombomere 1.

The MLF (medial longitudinal fasciculus) is one of the first tracts to form in the vertebrate brain and becomes a major conduit for fiber populations involved in motor coordination [39]. The MLF is a prototypical longitudinal tract, pioneered by a small group of axons that project in a precise position adjacent and parallel to the floor plate. To evaluate the position of the growing pioneers of the MLF with respect to Netrin1, the Netrin1 expression pattern in the E10 mouse mid- and hindbrain was visualized using in situ hybridization. Netrin1 expression domains include ventral midbrain around the cephalic flexure, intensely in the floor plate of the hindbrain, and a domain of lower expression extending away from the floor plate into the lateral neural tube (Figure 1A). The high floor plate and low lateral expression in the hindbrain closely resembles expression in the spinal cord [10,40].

The MLF pioneers were labeled with neuron-specific βIII-tubulin antibody, which showed that the nucleus of the MLF arises in the ventral fore- and midbrain within a region of strong Netrin1 expression. The MLF runs caudally (Figure 1B, arrow) through the midbrain and hindbrain near the Netrin1-positive floor plate. Expression analysis also suggested that the MLF axons were immunoreactive for DCC, the main attractive receptor in many systems [13]. In addition, the hindbrain floor plate expression of Netrin1 overlaps with expression of all three Slit genes [21], which together indicates that the MLF axons are exposed to high levels of both Netrin1 and Slits as they project longitudinally adjacent to the hindbrain floor plate.

### Medial longitudinal fasciculus axons deviate dorsally in Netrin1 mutants

**Figure 2:**
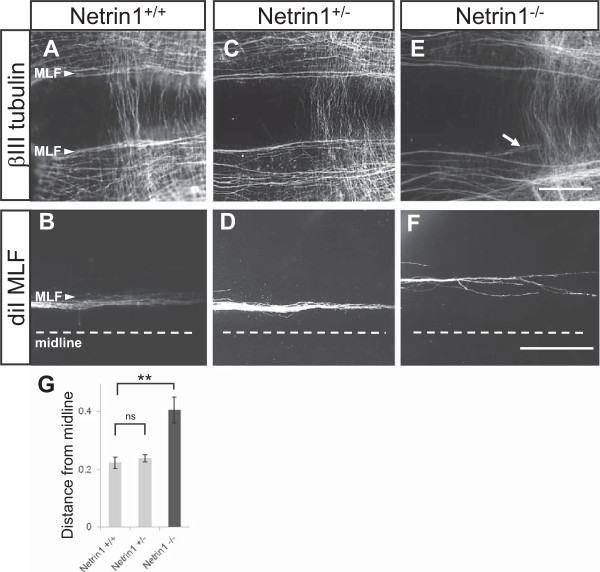
**Medial longitudinal fasciculus axons shift away from the midline in Netrin1 mutants.** Views of the medial longitudinal fasciculus (MLF) axons growing through hindbrains from mouse embryos, shown as open-book whole mounts of the anterior hindbrain, with the ventral midline in the middle. **(A,C,E)** MLF axons in Netrin1^+/+^, Netrin1^+/−^, and Netrin1^−/−^ embryos on embryonic day 10.5 visualized by whole-mount βIII-tubulin antibody labels. The pair of MLF bundles on either side of the midline are indicated by the arrow heads **(A)**. Netrin1^+/+^ and ^+/−^ embryos had normal MLF projections parallel to and close to the floor plate. **(E)** Netrin1^−/−^ embryos had aberrant MLF projections, including an increased distance from the floor plate, and truncated axon bundles (arrow) that split off from the main tract. **(B,D,F)** MLF axons labeled by a single crystal of the fluorescent axon tracer diI in the midbrain MLF nucleus in control and Netrin1^−/−^ embryos. Dashed lines mark the ventral midline, centered in the floor plate. **(G)** Quantification of MLF distance from the midline. The distance of the ventral-most MLF axons from the midline in diI-labeled embryos was normalized for embryo size to compensate for slightly different developmental stages (normalized distance = MLF distance/width of neural tube). (Quantification strategy is further described in Additional file 1). MLF axons were nearly twice as far away from the midline in Netrin1^−/−^ mutants (n = 7), compared to ^+/+^ (n = 5) and ^+/−^ (n = 7) embryos. Error bars show SEM; significance was measured using the *t*-test. **P < 0.01. ns, not significant. Scale bars in (E) and (F) indicate 200 μm, and apply to each row of images.

To test the role of Netrin1 in the guidance of longitudinal axons, Netrin1^−/−^ mutant mouse embryos were examined during the stage of active MLF outgrowth (embryonic day 10.5, E10.5) using whole-mount labeling with neuron-specific βIII-tubulin antibody (Figure 2). Overall, in Netrin1 mutants, the main fascicle of the MLF shifted away from the floor plate compared to wild-type and heterozygous control embryos. Bundles of axons deviated dorsally from the normal ventral position, leading to a wider gap between the bilateral MLF bundles. (Note the MLF errors occurred in conjunction with apparently normal trajectories of hindbrain commissural axons crossing the floor plate in Netrin1 mutants, suggesting that pre-crossing commissural axons are still attracted toward the ventral midline in the absence of Netrin1. We are addressing this issue in a separate study).

The aberrant MLF trajectories in Netrin1 mutants were also analyzed by diI tracing from the midbrain source of the MLF (Figure 2). In homozygous wild-type or heterozygous controls, the MLF formed a tight bundle of axons against the edge of the floor plate (Figure 2B,D). In Netrin1 mutants, the MLF axons had dorsal-angling trajectories, and diverged into multiple bundles. The axons in Netrin1 mutants traveled at a significantly increased distance from the floor plate, almost twice as far away from the midline as normal (Figure 2G; quantification strategy described in Methods, and in Additional file 1). In fact many MLF axons diverged farther than the ventral-most subset included in the quantification. The dorsal shift in the absence of Netrin1 suggests that Netrin1 acts as a ventral-ward attractive cue that keeps the MLF near the floor plate. In addition, the wider tract implies that Netrin1 loss causes a large increase in the variability (wandering) or fasciculation of MLF axons.

The shift in MLF position was also verified by DCC antibody labeling at an earlier stage, E9.5, which showed that the ventral-most DCC + axons shifted dorsally, and that the overall numbers of longitudinal pioneer neurons was normal in Netrin1 mutants (Additional file 2). The shift in MLF position was not the result of a wider floor plate, because the floor plate on E9.5 embryos retained the same width and expression of a floor plate marker (Additional file 3).An additional phenotype in Netrin1 mutant embryos was the appearance of prematurely truncated bundles of axons (Figure 2E). These bundles were clearly visible in both antibody and diI labels and occurred in both midbrain and hindbrain. All truncated bundles projected at ventral angles. This phenotype was observed in most Netrin1 mutant embryos but no heterozygous or wild-type embryos. These stalled axons suggest an additional role for Netrin1 in axon outgrowth promotion.

### Medial longitudinal fasciculus axons deviate dorsally in DCC mutants

**Figure 3:**
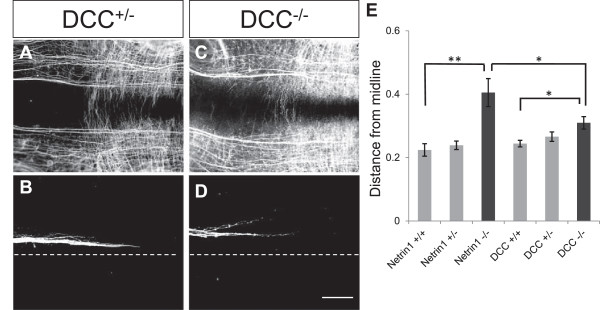
**Medial longitudinal fasciculus axons shift away from the midline in deleted in colorectal cancer receptor mutants. (A-D)** Deleted in colorectal cancer (DCC) mutant and control embryonic day 10.5 embryos labeled by whole-mount βIII-tubulin antibody **(A,C)** and diI labels **(B,D)**. **(E)** Axon positions were quantified, normalized for variations in embryo size, and compared to the Netrin1 data from Figure 3. Medial longitudinal fasciculus axons shifted dorsally in DCC^−/−^ mutants (n = 12), compared to controls (DCC^+/+^, n = 9; DCC^+/−^, n = 9), but significantly less than in Netrin1 mutants. Error bars show SEM; significance was measured using the *t*-test. *P < 0.05; **P < 0.01. Scale bar in **(D)** indicates 200 μm and applies to **(A-D)**.

To test which receptor was mediating Netrin1 responses in the longitudinal axons, we examined MLF projections in mice with mutations in the main attractive Netrin receptor, DCC.In DCC mutants, MLF axons made errors that were similar to Netrin1 mutants (Figure 3). MLF axons in DCC mutants shifted away from the floor plate. The average distance was significantly increased over controls, but was also significantly less than the Netrin1 mutants (Figure 3E). Some bundles deviated dorsally, creating bifurcations in the tract, although the total breadth of the tract appeared more compact than Netrin1 mutants. The difference between Netrin1 loss-of-function and DCC loss-of-function suggests that DCC mediates part but not all of the guidance activity of Netrin1.

### Medial longitudinal fasciculus axons shift toward the floor plate when Slit signals are reduced

The shifts of axons away from the ventral midline in Netrin1 or DCC mutants suggest that reduced midline attraction may cause sensitization to midline repellent signals. Slit1, 2, and 3 were good candidate repellents, because our prior study showed that Slit1 and 2 prevent MLF axons from entering and crossing the midbrain floor plate, at least in the midbrain where these are the only two Slits expressed during MLF pioneer navigation [18]. However, the hindbrain is potentially a different guidance environment, because Slit3 is expressed in combination with Slit1 and 2. Consistent with the potentially overlapping Slit functions in the hindbrain, Slit1/2 double mutants have less severe errors in this region, with some MLF axons crossing but few longitudinal projections within the floor plate itself [18]. These results suggested a midline inhibitory role for Slit1 and 2, but did not give a complete test of Slit function in the hindbrain due to the presence of Slit3. In addition, the previous analysis did not give insights into whether Slits were acting as local non-permissive cues to keep longitudinal axons out of the midline, or whether they acted as diffusible instructive cues to guide ipsilateral trajectories.

Therefore, we undertook an analysis of the genetic function of Slit3, in concert with Slit1 and 2, by analyzing a series of combinations of Slit1, 2, and 3 mutations. Triple homozygous mutants were reported in one prior publication [16].

**Figure 4:**
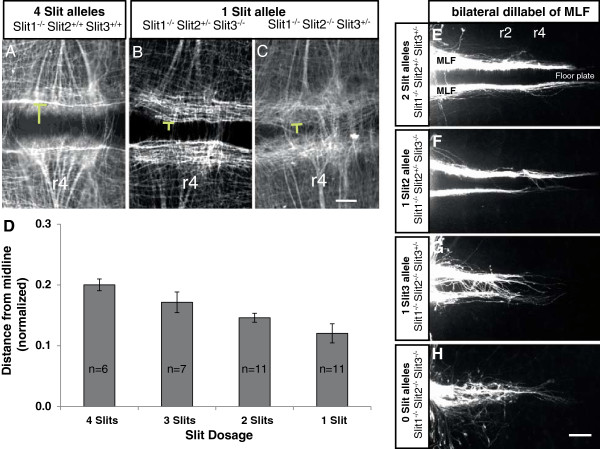
**Medial longitudinal fasciculus axons shift toward the midline in a Slit dose-dependent manner. (A-C)** Close-up views of the midline in rhombomere 4 (r4) in embryonic day 10.5 mouse embryos stained with βIII-tubulin antibody. The position (yellow line) of the most ventral medial longitudinal fasciculus (MLF) axons was quantified. **(A)** Embryos with four Slit doses as littermate controls for the normal MLF distance from midline in r4. **(B)** Slit mutants with only one remaining dose of Slit2 caused the most ventral MLF axons to shift toward the midline. **(C)** One dose of Slit3 caused a severe phenotype in which a subset of MLF axons had angling trajectories that crossed the midline. In these cases, subsets of axons which did not cross but maintained longitudinal trajectories were used to measure the MLF distance from midline. **(D)** Average normalized distance from the midline to the most ventral MLF fibers in r4 decreased gradually with loss of Slit doses. The number of embryos (n) is a sum of different combinations of Slit2 and 3 wild-type alleles, with 6 to 11 analyzed for each genotype (indicated on each bar). Error bars show SEM. Differences between genotypes were statistically significant by analysis of variance (*P* < 0.005). The trend in MLF position was significant by an ordered heterogeneity test (*P* < 0.0001) (see Methods). **(E-H)** Slit triple mutants. Bilateral diI tracing of MLF axons in littermates with decreasing Slit doses. Embryos with only one dose of either Slit2 or Slit 3 causes a subset of axons to cross the midline. Slit1/2/3 triple mutant embryos (n = 6) had a complete collapse of MLF axons into and projecting longitudinally within the floor plate. Scale bar in **(C)** also applies to **(A-C)**: 100 μm; scale bar in **(H)** also applies to **(E-H)**: 100 μm.

We reasoned that informative effects on longitudinal trajectories might result from partial reduction of Slit gene dosage, with the prediction that reduced Slit function would lead to a less repulsive floor plate, and cause MLF axons to shift ventrally. To test the effects of reducing Slit expression, we examined mouse embryos with different Slit allele doses (Figure 4). The genetic background of all lines was Slit1^−/−^, which on its own results in normal MLF projections [18]. By varying Slit 2 and 3 dosage, embryos were produced with one to four functional Slit alleles. Analysis of axon patterns was done primarily with whole-mount βIII-tubulin antibody labeling (Figure 4A-D), and axon patterns were further verified using specific diI tracing of the MLF (Figure 4E-H). Ventral shifts in MLF positions were evidenced by narrowing of the space between the flanking MLF bundles, with the strongest effects in rhombomere 4 (r4), where MLF positions were quantified. Reducing Slit dosage resulted in a graded narrowing of the distance between MLF bundles. Embryos with only one functional Slit allele had the largest average ventral shift of the MLF. Interestingly, Slit1^−/−^;2^+/−^;3^−/−^ embryos with a single Slit2 allele had MLF positions farther from the midline than in Slit1^−/−^;2^−/−^;3^+/−^ embryos, which was the most strongly affected genotype. Similar Slit dosage effects were seen in other hindbrain segments, though less dramatically than in r4 (not shown). Tracing of the MLF axons showed that midline crossing occurred in embryos with only 1 or 2 Slit alleles, and again a single Slit2 wild-type allele was more effective than a single Slit3 wild-type allele (Figure 4F,G).

**Figure 5:**
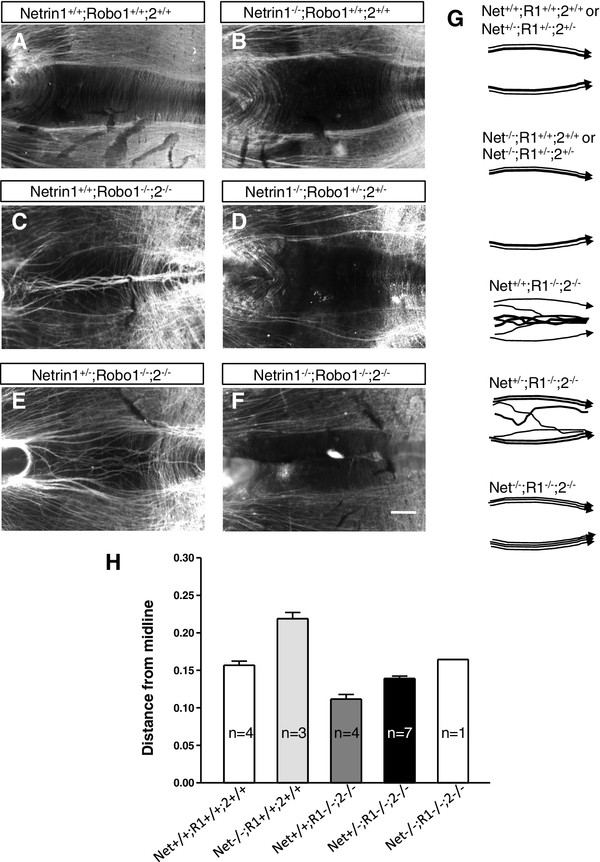
**A balance between Netrin and Slit/Robo signaling sets the in vivo position of medial longitudinal fasciculus axons.** Combined mutants between Netrin1, Robo1, and Robo2. **(A-F)** Whole-mount open book preparations stained with βIII-tubulin antibody. (Key genotypes with significant phenotypes are shown; several classes of heterozygous combinations did not cause medial longitudinal fasciculus (MLF) shifts, and are not shown). **(A-C)** Compared to the control MLF (wild type in **(A)**, n = 4), the MLF shifted away from the midline in Netrin1^−/−^ mutants (**B**, n = 3). In Robo1/2 mutants, the MLF shifted toward the midline and, in addition, many axons entered and grew longitudinally within the midline (**C**, n = 4). **(D)** When Robo1/2 function was reduced in a Netrin1 mutant background, Netrin1^−/−^; Robo1^+/−^;2^+/−^, the MLF maintained a similar dorsal shift (n = 1). **(E)** When Netrin1 function was reduced in a Robo1/2 mutant background, Netrin1^+/−^; Robo1^−/−^;2^−/−^, the MLF tracts shifted to a near-normal position, and the number of midline axon bundles was reduced (n = 7). (F) A single triple mutant embryo was isolated, showing an MLF pattern similar to wild-type, with neither dorsal or ventral shifts (n = 1). **(G)** Schematic summary of longitudinal pioneer patterns in key genotypes. **(H)** Graph of average normalized distance from the midline to the most ventral MLF fibers. All means differed significantly by analysis of variance (*P* < 10^−7^), and by pair-wise *t*-tests. Scale bar in **(F)** also applies to all: 100 μm.

Finally, Slit1/2/3 triple homozygotes had a strong shift of all MLF axons into the midline, with prominent longitudinal projections within the floor plate (Figure 4H). The shift of MLF axon trajectories into the midline is very similar to the axon errors in Robo1/2 double mutants (for example, see Figure 5C), supporting the idea that these two Robo receptors are the primary repulsive Slit receptors for longitudinal axons [18,27]. The graded shift in MLF positions caused by reducing Slit gene dosage implies that the three Slits act together to form a repulsive gradient to push MLF axons away from the midline.

### Medial longitudinal fasciculus trajectories in vivo are guided by a balance between Netrin1 and Slit/Robo signals

The analysis of Netrin/DCC and Slit/Robo guidance of the MLF pioneers suggests that the shifts in the MLF trajectories in these mutants are caused by an imbalance between attraction and repulsion. In Netrin1 or DCC mutants, the axons shift away from the floor plate, suggesting that repulsion is stronger than attraction. Conversely, the axons shift closer or into the floor plate in Slit or Robo mutants, suggesting an environment in which attraction is stronger than repulsion. However, while the opposing shifts caused by reducing either attraction or repulsion alone are consistent with a balancing mechanism between Netrin and Slit cues, we sought additional genetic evidence for the in vivo relevance of this balance. If the axons shift into the floor plate in Robo1/2 mutants because they are strongly attracted to Netrin1, then a reduction in Netrin1 attraction is predicted to suppress the shift of axons into the floor plate. Similarly, the shift of axons away from the floor plate in Netrin1 mutants should be alleviated by a reduction of Slit/Robo repulsion.

We took an in vivo genetic approach by attempting to make combined mutants between the two guidance systems, which have not previously been reported. Practical considerations guided us to make Netrin1; Robo1;2 triple mutants. We chose Netrin1 mutants to test attraction, because the homozygous Netrin1 mutant phenotype was stronger than DCC. For testing Slit/Robo repulsion, Robo1/2 mutants were used because the two genes are closely linked and segregate as one mutant allele, simplifying the genetic crosses. Importantly, genetic evidence suggests that Robo1 and 2 are the dominant Slit receptors for MLF axons, because the axons strongly enter the floor plate in Robo1/2 homozygous mutants [18,27], which is similar to Slit1/2/3 triple mutants (Figure 4).

When Netrin1 function was reduced in the Robo1/2 mutants (that is, Netrin1^+/−^; Robo1^−/−^;2^−/−^) both of the Robo1/2-dependent MLF axon phenotypes were less severe (Figure 5E). The remnant MLF shifted back nearly to the wild-type position, with larger, more prominent bundles. In addition, the longitudinal bundles within the midline were less prominent and involved fewer axons. This suggests that the MLF midline entry and ventral shift in Robo1/2 mutants is dependent on ventral-ward Netrin1 attraction.

We also attempted to reduce Robo1/2 function in Netrin1 homozygous mutants. Unfortunately, after many litters from crosses between triple heterozygotes producing a total of 99 embryos, the distribution of genotypes indicated a strong lethal genetic interaction between the Netrin1 and Robo1/2 mutant alleles. In the Netrin1^−/−^ background, when Robo1/2 gene dosage was reduced by half (that is, Netrin1^−/−^; Robo1^+/−^;2^+/−^) only one embryo was isolated (1/99), instead of a predicted 1/8 ratio. The low yield suggests a genetic requirement of all three genes for viability at an embryonic stage prior to E10.5. We also set up lines to combine Netrin mutations with either single Robo mutant (that is, Netrin1^−/−^; Robo1^+/−^ and Netrin1^−/−^; Robo2^+/−^) but these also produced lethal interactions because we were unable to isolate double homozygotes from either cross (not shown). We speculate that the Netrin/Robo lethal interaction may involve non-neuronal tissues, such as tissue movements during gastrulation [41,42], or early vascular or placental development [43-46]. Nonetheless, in the unique Netrin1^−/−^; Robo1^+/−^;2^+/−^ mutant embryo, the MLF retained a shift away from the midline similar to Netrin1^−/−^ mutants (Figure 5D). This result suggests that Robo1/2 heterozygotes retain sufficient Slit/Robo repulsion to push the MLF axons away from the midline.

The ultimate goal of combining Netrin1, Robo1, and Robo2 mutations also unfortunately resulted in only a single triple homozygote, Netrin1^−/−^; Robo1^−/−^;2^−/−^, identified on E10.5 (thus also 1/99 instead of a predicted 1/16). Strikingly, this remaining embryo had MLF tracts formed at positions close to wild-type (Figure 5F). No MLF axons entered or projected longitudinally within the floor plate. Thus, the Netrin1 mutant shift away from the midline was suppressed by removing Robo1/2 repulsive function. A complementary interpretation is that the Robo1/2 mutant shift toward and into the midline was suppressed by removing Netrin1 attractive function. The axon patterns are consistent with the partial restoration of MLF position in Netrin1^+/−^; Robo1^−/−^;2^−/−^ mutants. Together, these observations of combined Netrin1 and Robo1/2 mutants are consistent with a guidance mechanism in which Netrin1 and Slit/Robo signals act in balance to position the MLF axon trajectories.

As a side note, to attempt to bypass the triple mutant lethality, we also combined Netrin1 mutations with each individual Robo (that is, Netrin1^+/−^; Robo1^+/−^ crosses in a Robo2^+/+^ genetic background, and Netrin1^+/−^; Robo2^+/−^ crosses in a Robo1^+/+^ genetic background). Although we did not pursue this as exhaustively as for the triple mutants, no double homozygous mutants in either background were identified (not shown), suggesting the same lethal genetic interaction between Netrin1 and either Robo1 or Robo2.

### Medial longitudinal fasciculus axons can react directly in culture to combined Netrin1 and Slit signals

**Figure 6:**
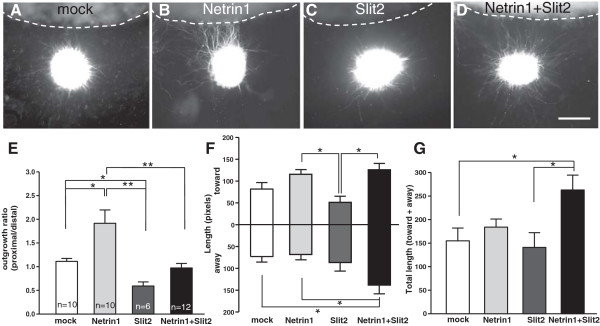
**Netrin1 and Slit2 have opposing effects on direction, and synergistic effects on growth, of explanted medial longitudinal fasciculus axons.** Tissue was dissected from ventral midbrain of embryonic day 11.5 mouse embryos, placed in collagen gel, and cultured for 48 hours with guidance cues. **(A-D)** Ventral midbrain explants were co-cultured with aggregates of (COS) cells transfected with no plasmid (mock, n = 10), Netrin1 (n = 10), or Slit2 (n = 6) expression plasmids. To combine Netrin and Slit signals, equal amounts of transfected cells were mixed to form aggregates (n = 12). Each explant is shown with the transfected COS aggregate at the top of the photograph (dashed lines). Axons were labeled with anti-deleted in colorectal cancer (DCC) antibodies, to avoid other nearby neuron types that may have been inadvertently included in the explanted tissue (for example, the DCC-negative oculomotor neurons). **(E)** Quantification of directional axon growth into quadrants toward or away from the COS cells. An outgrowth ratio was calculated by dividing the average axon length in pixels in the quadrant toward the cue source, divided by the quadrant away from the cue source. Note the neutral effects of untransfected COS cells, attraction by Netrin1 cells, and repulsion by Slit2 cells. Mixed aggregates secreting both Netrin1 and Slit2 had neutral directional effects, but caused a prominent increase in overall axon growth. The number of explants analyzed is shown on each bar, and also applies to graphs **(F)** and **(G)**. **(F)** Graph of average axon length in the quadrants toward and away from the cue source. **(G)** Graph of overall axon outgrowth, showing the sum from both quadrants. Note the significantly increased growth when Netrin1 and Slit2 signals were combined. Errors bars show ± SEM. Statistical comparison by *t*-test: *P < 0.05; **P < 0.01. Scale bar in **(D)** also applies to all: 100 μm.

The analysis of mutant mice indicates that Netrin and Slit signals have important genetic functions in positioning MLF axons in vivo. These results specifically imply that these longitudinal axons can respond to both attractive and repulsive signals simultaneously. However, the in vivo phenotypes could result from direct or indirect effects. To test for direct effects of isolated Netrin1 and Slit signals, we established an explant culture system for MLF axons. Small pieces of ventral midbrain tissue containing the nucleus of the MLF were embedded in collagen gels and cultured. Although specific molecular markers for MLF axons are not available, all axons growing out of the explants did label with DCC antibodies as expected for the MLF (Figure 1D; Figure 6).Ventral midbrain explants were co-cultured in the presence of COS cells transfected with secreted forms of Netrin1 or Slit2 (Figure 6). Axons cultured in the presence of control COS cell aggregates transfected with YFP had no directional influence. Netrin1-transfected COS cells had a positive effect on the direction of axon growth: axons grew toward Netrin1-expressing aggregates. In contrast, MLF axons had strong inhibitory responses to Slit2 because they grew away from Slit2-expressing aggregates. These effects suggest that MLF axons can respond directly to Netrin and Slit signals, with opposing responses consistent with their in vivo effects.To more closely reflect the situation in vivo, explants were cultured adjacent to COS aggregates secreting both Netrin1 and Slit2 (Figure 6). The presence of Netrin1 counteracted the repulsive effects of Slit2, resulting in symmetrical growth, which is evidence that MLF axons can simultaneously react to the two opposing signals. Surprisingly, the axons of explants exposed to an overlapping gradient of both Netrin1 and Slit2 were longer than those challenged with either single cue (Figure 6G). This indicates a synergistic effect of Slits and Netrin1 on MLF outgrowth, in addition to the individual instructive positive and negative signals. Together, the explant experiments indicate that Slits and Netrin1 cooperate to both guide and promote the outgrowth of MLF axons.

## Discussion

Longitudinal axon trajectories maintain precise positions relative to the DV axis of the neural tube, so rather than having one decision point at an intermediate target, longitudinal axons need continuous guidance input. Our results suggest that pioneer MLF trajectories are set by the combined action of Netrin1 and Slit signals. This navigation mechanism depends on a counter balance of attractive and repulsive cues, integrated simultaneously in a “push-pull” manner, to guide axons longitudinally at a specific position. MLF axon responses in culture further suggest that the combined signals synergistically increase the growth rate of these axons. The importance of a balance between these opposing cues is supported by several lines of evidence.

Our first major finding was that Netrin1 loss caused longitudinal axons to wander dorsally. Even the ventral-most axons in mutant embryos were significantly farther away from the floor plate than wild-type axons, and many axon trajectories diverged farther. Additionally, bundles with prematurely truncated axons indicate a growth-promoting function, which was further supported by observation of outgrowth towards a Netrin1 source in vitro. Netrin1 has at least two positive effects on MLF axons, which together serve to position and promote the growth of the MLF near the floor plate. A major Netrin receptor for longitudinal axons appears to be DCC, because DCC mutant axons shifted significantly away from the midline.

Slit signaling is also critically important for positioning longitudinal pioneer axons as an opposing repellent. Our previous study of Slit1/2 mutant mouse embryos showed that Slits are required to prevent MLF entry into and crossing of the floor plate in the midbrain [18]. Blocking midline entry could be accounted for by a local inhibitory role of Slits within floor plate tissue. Here, we extended these findings to show that reduced Slit gene dosage causes MLF axons to shift toward the floor plate. This observation implies that Slits can act not just locally but at a distance, and in a graded manner. As Slits can also act at a distance on axons in culture, together these results suggest that Slit signals position MLF axons by repelling them away from the ventral midline.

Since MLF axons respond to the Slits and Netrin1 simultaneously along their trajectory, we propose that a combination of repulsive and attractive cues position pioneer MLF axons to navigate within a narrow zone adjacent to the floor plate. MLF axons are pushed away from the floor plate by Slit signals, and pulled toward the floor plate by Netrin1 signals. This model explains why weakening either the positive or negative signals causes MLF shifts: releasing axons from the ventral attraction causes dorsal shifts driven by Slit repulsion, and releasing axons from the ventral repulsion causes ventral shifts driven by Netrin attraction. To formally test this mechanism, we combined mutations between Netrin1 and Robo1/2, which partially or wholly suppressed the axon errors caused by loss of either of the single pathways. The positive synergy of Slit and Netrin on outgrowth rates in vitro further indicates that MLF axons can respond simultaneously to both sets of cues, and consistent with the region near the floor plate providing a growth-promoting environment for the rapidly advancing MLF axons. Another important implication is that silencing via a Robo-DCC hierarchy does not appear to occur in longitudinal axons. The key prediction of the silencing model is that if Slit/Robo signaling silences DCC-mediated attraction, then the presence or absence of DCC (or Netrin1) should have no effect on MLF guidance. However, mutants for either Netrin1 or DCC have major MLF shifts, showing that Netrin1/DCC signaling is active and critical for MLF navigation, despite the fact that they are growing next to the floor plate where they are exposed to the highest possible levels of Slit repulsion.

The mechanism of longitudinal guidance by balanced Netrin and Slit cues in mouse embryos extends, in several ways, a previous report on the guidance of longitudinal axons through the zebrafish hindbrain [20]. That study showed that a different longitudinal axon population, which is dopaminergic and descends through the hindbrain at a more dorsal position, shifted toward the ventral midline in Robo2/astray mutants [20], consistent with Robo guidance being critical to keep mouse longitudinal axons out of the midline [18]. However, single knockdowns of either zebrafish Netrin, DCC, or combinations of the Slits did not perturb longitudinal guidance in a wild type genetic background [20]. The zebrafish experiments did suggest that Netrin/DCC and Slit/Robo signaling was involved in the Robo2-dependent positioning of tracts, because Netrin or DCC morpholino knockdown in Robo2 mutants was effective at suppressing the mutant shift toward the midline. Our results provide a more comprehensive test of how these cues guide the mouse MLF, because mutants for Netrin1, DCC, or the Slits do have longitudinal shifts, and explants also confirmed that the cues can act directly on cultured axons. The absence of zebrafish phenotypes for several of the key molecules may be due to partial knockdown via morpholinos, which agrees with our observations that heterozygous mouse embryos maintained normal tract positions. However, it should also be noted that the zebrafish mutants or knockdowns perturbed only a specific dopaminergic axon subpopulation, while strikingly the zebrafish MLF axons appeared to project normally [20]. The comparison of these mouse and zebrafish studies emphasizes that a good understanding of longitudinal guidance mechanisms will require additional axon populations to be studied in a variety of experimental approaches.

An interesting implication of the push-pull mechanism is that it provides a general strategy for DV positioning of axon populations, where increased relative attraction to Netrin1 could override Slit repulsion to result in a ventral position, or increased repulsive responses to Slits would result in more dorsal positions. For example, increases in Robo3 expression appear to interfere with Robo-mediated repulsion to cause shifts in zebrafish longitudinal tract positions [47]. However, evidence suggests that shifts in axon responses are unlikely to be simply regulated by receptor levels on axons or growth cones. On one hand, MLF axons show the brightest DCC labeling of the pioneer descending tracts, which could mediate strong attraction ventrally. On the other hand, Robo1 expression also appears highest in MLF [18,27], so the position of the MLF as the ventral-most tract is not simply explained by an imbalance of attractive receptors over repulsive receptors. This suggests that receptor signaling may be modulated in other ways such as cell surface localization or downstream signaling modulators.

Longitudinal navigation involves the integration of positive and negative cues that are opposed, and potentially confusing. Binding of either Slits or Netrin to their receptors sets off a cascade of intercellular events that affect multiple pathways [48,49]. Interestingly, Netrin and Slit signal transduction pathways have opposing effects on common components such as the small GTPase Cdc42 [50,51]. Other potential switches between attraction and repulsion include cyclic nucleotide levels and calcium levels [52]. Thus, converging signals could be integrated to provide a fine-tuned response to opposing Slit and Netrin signals. Future experiments on receptor signaling mechanisms and the cell biology of growth cones will be needed to reveal integration mechanisms for longitudinal guidance.

The restoration of a normal MLF projection pattern in the combined Netrin1/Robo1/2 triple mutant is an intriguing example of the ability of axons to continue to navigate, even in a “ground-state”, devoid of Netrin1/Slit guidance. That the axons can overcome the loss of their main guidance cues suggests that additional signals likely also play roles in longitudinal navigation. These may include other midline attractants such as Sonic hedgehog (Shh) or vascular endothelial growth factor (VEGF) [33,34], midline repellents such as Semaphorins [53], and directional cues along the anterior-posterior axis such as Wnts or Shh [54-57]. Axon-axon interactions also play a role in regulating the cohesion of longitudinal tracts [19]. Further evidence that long range cues can act by modulating axonal expression of adhesion molecules suggest important interactions between long range and local guidance mechanisms [53].

Studies from a range of systems suggest that Netrin and Slit cues can be interpreted by axons in diverse ways. In fact, the initial nematode Netrin mutants were identified by errors in circumferential axons [28], which grow away from Slit and toward Netrin, implying both cues act simultaneously and cooperatively on these axons [29,58,59]. In *Drosophila*, Slit and Netrin cues are secreted together from the midline, but genetic analysis suggests that they signal in parallel in an additive manner, rather than in a hierarchical silencing mechanism [31]. Other systems have hierarchical cue interactions which are quite distinct from silencing. For example, mouse thalamocortical axons are repelled when presented by Slits alone, and do not react to Netrin1 alone, but Slit switches to attraction in the presence of Netrin1 to promote projections into the rostral cortex [37]. This switch in thalamocortical responses requires a novel Robo1 co-receptor, FLRT3, which acts to upregulate DCC as an attractive Netrin1 receptor [60], but it remains unknown whether these receptor interactions have wider roles in other axon populations. Netrin and Slit cues interact differently in the mouse corpus callosum, where neocortical axons again do not react to Netrin1 alone, but Netrin1/DCC signaling instead attenuates Slit repulsion, allowing midline crossing [38]. Similarly, DCC inhibits Slit/Robo repulsion of precrossing axons in zebrafish spinal cord, allowing them to approach the midline [61]. Together, the diversity of potential axonal reactions to just this pair of cues is impressive, as their interactions can be hierarchical in either direction, cause switching of repulsion to attraction, or can be balanced in a non-hierarchical fashion, as we show for longitudinal pioneers. It seems likely that additional novel interactions between axon guidance signals are critical for axon circuit formation.

## Conclusions

Our evidence indicates that longitudinal axons navigate using simultaneous Netrin1/DCC and Slit/Robo signals to set their trajectories. This push-pull mechanism suggests that longitudinal axons integrate the opposing signals through a balance that constrains their growth into longitudinal trajectories at specific positions. Overall, our results build upon the model that the molecular landscape of guidance cues laid out in embryonic brain tissue is interpreted in various ways by different types of axons to generate diverse and specific patterns of tracts to wire the brain.

## Methods

### In situ hybridization

Whole mount in situ hybridization was carried out using standard procedures [62]. Probes for Netrin1 and Slit1 were provided by Marc Tessier-Lavigne (Rockefeller University, New York, NY). DCC probes were generated by reverse transcription-PCR, using procedures previously described [63].

### Immunohistochemistry

Neural tubes were dissected from the rest of the embryo, and washed for several hours in PBS containing 10% FBS and 1% TritonX (PBT + serum). Primary antibody in PBT + serum was applied for 1 to 3 days: rabbit anti-βIII tubulin (Covance, Princeton, New Jersey; 1:1000), goat anti-DCC (Santa Cruz, Dallas, TX; 1:250). After washing in PBT + serum overnight, secondary antibodies (Jackson ImmunoLabs, West Grove, PA) were applied in PBT + serum at 1:200 for 1 to 2 days, followed by overnight washes.

### Mouse embryos

Animal experiment protocol #00435 was approved by the University of Nevada, Reno, IACUC, following guidelines of the National Institutes of Health, and accredited by the Association for Assessment and Accreditation of Laboratory Animal Care International. Expression analyses were performed with CD1 embryos. Netrin mutants were previously described, and genotyped by a combination of PCR amplification of the lacZ gene trap insert, intensity of Xgal labeling of embryonic spinal cord tissue (see Additional file 4), or examination of commissure formation in the embryonic spinal cord [64]. The Netrin1^−/−^ hindbrain floor plate appeared normal using the 4C7 antibody (DSHB) against HNF3b/FoxA2. DCC mutants were previously described, and genotyped by PCR [13]. Slit1^−/−^; Slit2^+/−^; Slit3^+/−^ triple mutant founder mice were a gift from Marc Tessier-Lavigne, (Rockefeller University) [15,16,65], and were mated to produce embryos with Slit1^−/−^ genotypes combined with various doses of Slit2 and Slit3.

To obtain Netrin1/Robo1/Robo2 triple mutants, Netrin1^+/−^ heterozygotes were first mated with mice carrying the linked Robo1/2 heterozygous mutations, and then the resulting Netrin1^+/−^; Robo1^+/−^; Robo2^+/−^ triple heterozygotes were intercrossed to maintain the new strain and to generate embryos. The embryos were genotyped by PCR of both Robo1 and 2 alleles, combined with histological examination of commissure formation in the embryonic spinal cord to verify the Netrin1 genotype. It is unlikely that the rarity of the triple mutant combination (1/99) was caused by a general vascular defect, because the embryos did not appear hypoxic within our triple het litters of embryos, and no “moles” were included in our analysis. Second, somites were counted for all of the embryos analyzed, and were within the expected range of development.

Embryos were collected on E10 or E10.5, with noon of the day of the vaginal plug designated as E0.5. The lipophilic fluorescent axon tracer DiI was used as previously described [5,66] to label the midbrain nucleus of the MLF and trace MLF axon trajectories as they descended through the hindbrain.

### Quantification of normalized distance from the midline

The distance of the ventral-most axons from the midline in DiI and βIII-tubulin labels was measured at regular intervals along the length of the neural tube using ImageJ from NIH (Bethesda, MD; see Additional file 1). For the Netrin analysis, to normalize for embryo size, distance from the midline was divided by the total DV width of the neural tube as measured at the boundary between r1 and r2. The average normalized distance for each embryo was then used to perform statistical analysis by analysis of variance (ANOVA), which found that the means were significantly different, and by *t*-test.

For the analysis of MLF position in Slit mutants, measurements between the MLF fascicles of βIII-tubulin labeled embryos were halved to represent average distance from the midline, and expressed as ratios as above. The numbers were pooled for Slit wild-type allele dose, ranging from 4 to 1. The four groups of means were compared by ANOVA, which found that the means were significantly different at *P* < 0.005. As another statistical comparison, the decreasing trend of the means as Slit doses decrease from 4 to 1 was analyzed by an ordered heterogeneity test [67], in which the expected rank order was found to be the same as the observed rank order, with a confidence level of *P* < 0.0001.

The MLF position in Netrin1/Robo1/Robo2 triple mutants was quantified by the same strategy, and the means for different genotypes were found to be significantly different by ANOVA (*P* < 10^−6^), and by appropriate pairs of *t*-tests.

### Explant co-culture

Ventral mid- and forebrain tissue of E11.5 embryos was dissected to include the source of the MLF, and to exclude the medial-most tissue containing motor neurons of nIII. Culture conditions for explants were as described previously [33]. To provide localized Netrin1 or Slit sources, COS cells were transfected with a chick Netrin1 expression plasmid (gift of Marc Tessier-Lavigne, Rockefeller) [11] or a human Slit2 expression plasmid (gift of Yi Rao, Peking (Beijing, China)) [68]. COS cells were cultured for 24 hours, then aggregates formed and placed near tissue explants. Combined Netrin1 and Slit2 signals were provided by mixing equal amounts of cells transfected with each plasmid. Expression of the cues was verified using anti-myc antibodies to label fixed COS cell cultures. Explants were cultured for 48 hours, and then fixed. Anti-DCC or βIII-tubulin antibody was used to visualize MLF axons. The length of axons was measured using the ImageJ plugin NeuronJ to determine mean axon lengths from each explant, with the number of axons ranging from 7 to 62. For directional analysis, quadrants were marked on images using Adobe Illustrator (San Jose, CA), then imported and quantitated in Image J.

## Abbreviations

ANOVA: analysis of variance; COS: CV-1 (simian) in Origin, and carrying the SV40 genetic material; DCC: deleted in colorectal cancer; DV: dorsal-ventral; E: embryonic day; FBS: fetal bovine serum; MLF: medial longitudinal fasciculus; PBS: phosphate-buffered saline; PBT: PBS containing 1% TritonX; PCR: polymerase chain reaction; r4: rhombomere 4; Shh: Sonic hedgehog; VEGF: Vascular endothelial growth factor.

## Competing interests

The authors declare that they have no competing interests.

## Author’ contributions

MK and WTF contributed equally to the manuscript, and are listed as co-first authors. MK, WTF, BB, and SAM contributed to the conception/design of the study, the acquisition, analysis, and interpretation of data, and edited the manuscript. PJF contributed to the acquisition of data and edited the manuscript. FC contributed to the conception/design of the study, interpretation of data, and edited the manuscript. GSM contributed to the conception/design of the study, the acquisition, analysis, and interpretation of data, and edited the manuscript. All authors read and approved the final manuscript.

## Supplementary Material

Additional file 1 **Quantification of MLF distance from the midline.** Example showing the measurements made to compare the distance of MLF axons from the midline. Differences in developmental stage were normalized relative to the width (large bracket, wr1r2) of neural tissue at the r1/r2 border. Click here for file

Additional file 2 **Netrin1 mutant longitudinal axons retain wild-type axon numbers and DCC expression.** (A-C) Whole mount embryos on E9.5 labeled with DCC antibody. The DCC labeling intensity was consistently lower in Netrin1^+/+^ control embryos. (D) Quantification of numbers of longitudinal axons on E9.5, by counting longitudinal axons on each side of the hindbrain in images of whole mounts. The n numbers indicate the number of embryos analyzed for each genotype; the error bars indicate SEM. By ANOVA analysis, there were no significant differences in axon number between the genotypes. Both ventral and dorsal axons were included in these counts. The size of the most ventral bundle, consisting of the MLF axons, was similar between genotypes, but tight fascicles made it difficult to determine the number of individual axons. Scale bar: 100 μm. Click here for file

Additional file 3 **Netrin1 mutants retain normal hindbrain floor plate size and specification.** Sections through E9.5 hindbrain labeled with the 4C7antibody against HNF3b/FoxA2, a transcription factor expressed in a domain including the floor plate and adjacent ventral cells. The morphology and size of the floor plate domain appears similar in controls and Netrin1 mutants. Scale bar: 100 μm. Click here for file

Additional file 4 **Genotyping of Netrin1 mutant embryos.** Beta-galactosidase antibody labeling of spinal cord sections. Note the higher intensity of antibody labeling in homozygous mutants. Scale bar: 100 μm. Click here for file

## References

[B1] Chitnis AB Kuwada JY Axonogenesis in the brain of zebrafish embryos J Neurosci 1990 10 1892 1905 2355256 10.1523/JNEUROSCI.10-06-01892.1990 PMC6570297

[B2] Chedotal A Pourquie O Sotelo C Initial tract formation in the brain of the chick embryo: selective expression of the BEN/SC1/DM-GRASP cell adhesion molecule Eur J Neurosci 1995 7 198 212 7757257 10.1111/j.1460-9568.1995.tb01056.x

[B3] Wilson SW Ross LS Parrett T Easter SS Jr The development of a simple scaffold of axon tracts in the brain of the embryonic zebrafish, Brachydanio rerio Development 1990 108 121 145 2351059 10.1242/dev.108.1.121

[B4] Easter SS Ross LS Frankfurter A Initial tract formation in the mouse brain J Neurosci 1993 13 285 299 8423474 10.1523/JNEUROSCI.13-01-00285.1993 PMC6576300

[B5] Mastick GS Easter SS Initial organization of neurons and tracts in the embryonic mouse fore- and midbrain Dev Biol 1996 173 79 94 8575640 10.1006/dbio.1996.0008

[B6] Ware M Schubert FR Development of the early axon scaffold in the rostral brain of the chick embryo J Anat 2011 219 203 216 21599661 10.1111/j.1469-7580.2011.01389.x PMC3162240

[B7] Colamarino SA Tessier-Lavigne M The role of the floor plate in axon guidance Annu Rev Neurosci 1995 18 497 529 7605072 10.1146/annurev.ne.18.030195.002433

[B8] Kaprielian Z Runko E Imondi R Axon guidance at the midline choice point Dev Dyn 2001 221 154 181 11376484 10.1002/dvdy.1143

[B9] Evans TA Bashaw GJ Axon guidance at the midline: of mice and flies Curr Opin Neurobiol 2010 20 79 85 20074930 10.1016/j.conb.2009.12.006 PMC4128228

[B10] Kennedy TE Serafini T de la Torre JR Tessier-Lavigne M Netrins are diffusible chemotropic factors for commissural axons in the embryonic spinal cord Cell 1994 78 425 435 8062385 10.1016/0092-8674(94)90421-9

[B11] Serafini T Kennedy TE Galko MJ Mirzayan C Jessell TM Tessier-Lavigne M The netrins define a family of axon outgrowth-promoting proteins homologous to C. elegans UNC-6 Cell 1994 78 409 424 8062384 10.1016/0092-8674(94)90420-0

[B12] Deiner MS Kennedy TE Fazeli A Serafini T Tessier-Lavigne M Sretavan DW Netrin-1 and DCC mediate axon guidance locally at the optic disc: loss of function leads to optic nerve hypoplasia Neuron 1997 19 575 589 9331350 10.1016/s0896-6273(00)80373-6

[B13] Fazeli A Dickinson SL Hermiston ML Tighe RV Steen RG Small CG Stoeckli ET Keino-Masu K Masu M Rayburn H Simons J Bronson RT Gordon JI Tessier-Lavigne M Weinberg RA Phenotype of mice lacking functional Deleted in colorectal cancer (Dcc) gene Nature 1997 386 796 804 9126737 10.1038/386796a0

[B14] Brose K Bland KS Wang KH Arnott D Henzel W Goodman CS Tessier-Lavigne M Kidd T Slit proteins bind Robo receptors and have an evolutionarily conserved role in repulsive axon guidance Cell 1999 96 795 806 10102268 10.1016/s0092-8674(00)80590-5

[B15] Bagri A Marín O Plump AS Mak J Pleasure SJ Rubenstein JL Tessier-Lavigne M Slit proteins prevent midline crossing and determine the dorsoventral position of major axonal pathways in the mammalian forebrain Neuron 2002 33 233 248 11804571 10.1016/s0896-6273(02)00561-5

[B16] Long H Sabatier C Ma L Plump A Yuan W Ornitz DM Tamada A Murakami F Goodman CS Tessier-Lavigne M Conserved roles for Slit and Robo proteins in midline commissural axon guidance Neuron 2004 42 213 223 15091338 10.1016/s0896-6273(04)00179-5

[B17] Kidd T Bland KS Goodman CS Slit is the midline repellent for the robo receptor in Drosophila Cell 1999 96 785 794 10102267 10.1016/s0092-8674(00)80589-9

[B18] Farmer WT Altick AL Nural HF Dugan JP Kidd T Charron F Mastick GS Pioneer longitudinal axons navigate using floor plate and Slit/Robo signals Development 2008 135 3643 3653 18842816 10.1242/dev.023325 PMC2768610

[B19] Devine CA Key B Robo-Slit interactions regulate longitudinal axon pathfinding in the embryonic vertebrate brain Dev Biol 2008 313 371 383 18061159 10.1016/j.ydbio.2007.10.040

[B20] Kastenhuber E Kern U Bonkowsky JL Chien CB Driever W Schweitzer J Netrin-DCC, Robo-Slit, and heparan sulfate proteoglycans coordinate lateral positioning of longitudinal dopaminergic diencephalospinal axons J Neurosci 2009 29 8914 8926 19605629 10.1523/JNEUROSCI.0568-09.2009 PMC4404407

[B21] Farmer WT Altick AL Dugan JP Kidd T Charron F Mastick GS Pioneer longitudinal axons navigate using floor plate and Slit/Robo signals Int J Dev Neurosci 2008 26 884 10.1242/dev.023325 PMC2768610 18842816

[B22] Molle KD Chédotal A Rao Y Lumsden A Wizenmann A Local inhibition guides the trajectory of early longitudinal tracts in the developing chick brain Mech Dev 2004 121 143 156 15037316 10.1016/j.mod.2003.12.005

[B23] Simpson JH Kidd T Bland KS Goodman CS Short-range and long-range guidance by slit and its Robo receptors. Robo and Robo2 play distinct roles in midline guidance Neuron 2000 28 753 766 11163264 10.1016/s0896-6273(00)00151-3

[B24] Rajagopalan S Vivancos V Nicolas E Dickson BJ Selecting a longitudinal pathway: Robo receptors specify the lateral position of axons in the Drosophila CNS Cell 2000 103 1033 1045 11163180 10.1016/s0092-8674(00)00207-5

[B25] Spitzweck B Brankatschk M Dickson BJ Distinct protein domains and expression patterns confer divergent axon guidance functions for Drosophila Robo receptors Cell 2010 140 409 420 20144763 10.1016/j.cell.2010.01.002

[B26] Jaworski A Long H Tessier-Lavigne M Collaborative and specialized functions of Robo1 and Robo2 in spinal commissural axon guidance J Neurosci 2010 30 9445 9453 20631173 10.1523/JNEUROSCI.6290-09.2010 PMC6632452

[B27] Kim M Roesener AP Mendonca PR Mastick GS Robo1 and Robo2 have distinct roles in pioneer longitudinal axon guidance Dev Biol 2011 358 181 188 21820427 10.1016/j.ydbio.2011.07.025 PMC3171630

[B28] Hedgecock EM Culotti JG Hall DH The unc-5, unc-6, and unc-40 genes guide circumferential migrations of pioneer axons and mesodermal cells on the epidermis in C. elegans Neuron 1990 4 61 85 2310575 10.1016/0896-6273(90)90444-k

[B29] Ren XC Kim S Fox E Hedgecock EM Wadsworth WG Role of netrin UNC-6 in patterning the longitudinal nerves of Caenorhabditis elegans J Neurobiol 1999 39 107 118 10213457

[B30] Harris R Sabatelli LM Seeger MA Guidance cues at the Drosophila CNS midline: identification and characterization of two Drosophila Netrin/UNC-6 homologs Neuron 1996 17 217 228 8780646 10.1016/s0896-6273(00)80154-3

[B31] Garbe DS Bashaw GJ Independent functions of Slit-Robo repulsion and Netrin-Frazzled attraction regulate axon crossing at the midline in Drosophila J Neurosci 2007 27 3584 3592 17392474 10.1523/JNEUROSCI.0301-07.2007 PMC6672108

[B32] Andrews GL Tanglao S Farmer WT Morin S Brotman S Berberoglu MA Price H Fernandez GC Mastick GS Charron F Kidd T Dscam guides embryonic axons by Netrin-dependent and -independent functions Development 2008 135 3839 3848 18948420 10.1242/dev.023739 PMC2712571

[B33] Charron F Stein E Jeong J McMahon AP Tessier-Lavigne M The morphogen sonic hedgehog is an axonal chemoattractant that collaborates with netrin-1 in midline axon guidance Cell 2003 113 11 23 12679031 10.1016/s0092-8674(03)00199-5

[B34] Ruiz De Almodovar C Fabre PJ Knevels E Coulon C Segura I Haddick PC Aerts L Delattin N Strasser G Oh WJ Lange C Vinckier S Haigh J Fouquet C Gu C Alitalo K Castellani V Tessier-Lavigne M Chedotal A Charron F Carmeliet P VEGF mediates commissural axon chemoattraction through its receptor Flk1 Neuron 2011 70 966 978 21658588 10.1016/j.neuron.2011.04.014 PMC3638787

[B35] Stein E Tessier-Lavigne M Hierarchical organization of guidance receptors: silencing of netrin attraction by slit through a Robo/DCC receptor complex Science 2001 291 1928 1938 11239147 10.1126/science.1058445

[B36] Fujisawa K Wrana JL Culotti JG The slit receptor EVA-1 coactivates a SAX-3/Robo mediated guidance signal in C. elegans Science 2007 317 1934 1938 17901337 10.1126/science.1144874

[B37] Bielle F Marcos-Mondéjar P Leyva-Díaz E Lokmane L Mire E Mailhes C Keita M García N Tessier-Lavigne M Garel S López-Bendito G Emergent growth cone responses to combinations of Slit1 and Netrin 1 in thalamocortical axon topography Curr Biol 2011 21 1748 1755 22000108 10.1016/j.cub.2011.09.008

[B38] Fothergill T Donahoo AL Douglass A Zalucki O Yuan J Shu T Goodhill GJ Richards LJ Netrin-DCC signaling regulates corpus callosum formation through attraction of pioneering axons and by modulating slit2-mediated repulsion Cereb Cortex 2013 24 1138 1151 23302812 10.1093/cercor/bhs395

[B39] Frohman TC Galetta S Fox R Solomon D Straumann D Filippi M Zee D Frohman EM Pearls & Oy-sters: The medial longitudinal fasciculus in ocular motor physiology Neurology 2008 70 e57 e67 18427066 10.1212/01.wnl.0000310640.37810.b3

[B40] Kennedy TE Wang H Marshall W Tessier-Lavigne M Axon guidance by diffusible chemoattractants: a gradient of netrin protein in the developing spinal cord J Neurosci 2006 26 8866 8874 16928876 10.1523/JNEUROSCI.5191-05.2006 PMC6674364

[B41] Yeo SY Little MH Yamada T Miyashita T Halloran MC Kuwada JY Huh TL Okamoto H Overexpression of a slit homologue impairs convergent extension of the mesoderm and causes cyclopia in embryonic zebrafish Dev Biol 2001 230 1 17 11161558 10.1006/dbio.2000.0105

[B42] Mawdsley DJ Cooper HM Hogan BM Cody SH Lieschke GJ Heath JK The Netrin receptor Neogenin is required for neural tube formation and somitogenesis in zebrafish Dev Biol 2004 269 302 315 15081375 10.1016/j.ydbio.2004.02.001

[B43] Park KW Crouse D Lee M Karnik SK Sorensen LK Murphy KJ Kuo CJ Li DY The axonal attractant Netrin-1 is an angiogenic factor Proc Natl Acad Sci U S A 2004 101 16210 16215 15520390 10.1073/pnas.0405984101 PMC528958

[B44] Wilson BD Ii M Park KW Suli A Sorensen LK Larrieu-Lahargue F Urness LD Suh W Asai J Kock GA Thorne T Silver M Thomas KR Chien CB Losordo DW Li DY Netrins promote developmental and therapeutic angiogenesis Science 2006 313 640 644 16809490 10.1126/science.1124704 PMC2577078

[B45] Liao WX Wing DA Geng JG Chen DB Perspectives of SLIT/ROBO signaling in placental angiogenesis Histol Histopathol 2010 25 1181 1190 20607660 10.14670/HH-25.1181 PMC8900672

[B46] Xie H Zou L Zhu J Yang Y Effects of netrin-1 and netrin-1 knockdown on human umbilical vein endothelial cells and angiogenesis of rat placenta Placenta 2011 32 546 553 21570114 10.1016/j.placenta.2011.04.003

[B47] Schweitzer J Löhr H Bonkowsky JL Hübscher K Driever W Sim1a and Arnt2 contribute to hypothalamo-spinal axon guidance by regulating Robo2 activity via a Robo3-dependent mechanism Development 2013 140 93 106 23222439 10.1242/dev.087825 PMC4497291

[B48] Patel BN Van Vactor DL Axon guidance: the cytoplasmic tail Curr Opin Cell Biol 2002 14 221 229 11891122 10.1016/s0955-0674(02)00308-3

[B49] Round J Stein E Netrin signaling leading to directed growth cone steering Curr Opin Neurobiol 2007 17 15 21 17254765 10.1016/j.conb.2007.01.003

[B50] Wong K Ren XR Huang YZ Xie Y Liu G Saito H Tang H Wen L Brady-Kalnay SM Mei L Wu JY Xiong WC Rao Y Signal transduction in neuronal migration: roles of GTPase activating proteins and the small GTPase Cdc42 in the Slit-Robo pathway Cell 2001 107 209 221 11672528 10.1016/s0092-8674(01)00530-x

[B51] Shekarabi M Kennedy TE The netrin-1 receptor DCC promotes filopodia formation and cell spreading by activating Cdc42 and Rac1 Mol Cell Neurosci 2002 19 1 17 11817894 10.1006/mcne.2001.1075

[B52] Nishiyama M Hoshino A Tsai L Henley JR Goshima Y Tessier-Lavigne M Poo MM Hong K Cyclic AMP/GMP-dependent modulation of Ca2+ channels sets the polarity of nerve growth-cone turning Nature 2003 423 990 995 12827203 10.1038/nature01751

[B53] Wolman MA Regnery AM Becker T Becker CG Halloran MC Semaphorin3D regulates axon interactions by modulating levels of L1 cell adhesion molecule J Neurosci 2007 27 9653 9663 17804626 10.1523/JNEUROSCI.1741-07.2007 PMC6672970

[B54] Lyuksyutova AI Lu CC Milanesio N King LA Guo N Wang Y Nathans J Tessier-Lavigne M Zou Y Anterior-posterior guidance of commissural axons by Wnt-frizzled signaling Science 2003 302 1984 1988 14671310 10.1126/science.1089610

[B55] Yam PT Kent CB Morin S Farmer WT Alchini R Lepelletier L Colman DR Tessier-Lavigne M Fournier AE Charron F 14-3-3 proteins regulate a cell-intrinsic switch from sonic hedgehog-mediated commissural axon attraction to repulsion after midline crossing Neuron 2012 76 735 749 23177959 10.1016/j.neuron.2012.09.017

[B56] Liu Y Shi J Lu CC Wang ZB Lyuksyutova AI Song XJ Zou Y Ryk-mediated Wnt repulsion regulates posterior-directed growth of corticospinal tract Nat Neurosci 2005 8 1151 1159 16116452 10.1038/nn1520

[B57] Bourikas D Pekarik V Baeriswyl T Grunditz A Sadhu R Nardo M Stoeckli ET Sonic hedgehog guides commissural axons along the longitudinal axis of the spinal cord Nat Neurosci 2005 8 297 304 15746914 10.1038/nn1396

[B58] Wadsworth WG Bhatt H Hedgecock EM Neuroglia and pioneer neurons express UNC-6 to provide global and local netrin cues for guiding migrations in C. elegans Neuron 1996 16 35 46 8562088 10.1016/s0896-6273(00)80021-5

[B59] Hao JC Yu TW Fujisawa K Culotti JG Gengyo-Ando K Mitani S Moulder G Barstead R Tessier-Lavigne M Bargmann CI C. elegans slit acts in midline, dorsal-ventral, and anterior-posterior guidance via the SAX-3/Robo receptor Neuron 2001 32 25 38 11604136 10.1016/s0896-6273(01)00448-2

[B60] Leyva-Díaz E Del Toro D Menal MJ Cambray S Susín R Tessier-Lavigne M Klein R Egea J López-Bendito G FLRT3 is a Robo1-interacting protein that determines Netrin-1 attraction in developing axons Curr Biol 2014 24 494 508 24560577 10.1016/j.cub.2014.01.042

[B61] Bonner J Letko M Nikolaus OB Krug L Cooper A Chadwick B Conklin P Lim A Chien CB Dorsky RI Midline crossing is not required for subsequent pathfinding decisions in commissural neurons Neural Dev 2012 7 18 22672767 10.1186/1749-8104-7-18 PMC3507651

[B62] Mastick GS Davis NM Andrew GL Easter SS Jr Pax-6 functions in boundary formation and axon guidance in the embryonic mouse forebrain Development 1997 124 1985 1997 9169845 10.1242/dev.124.10.1985

[B63] Nural HF Todd Farmer W Mastick GS The Slit receptor Robo1 is predominantly expressed via the Dutt1 alternative promoter in pioneer neurons in the embryonic mouse brain and spinal cord Gene Expr Patterns 2007 7 837 845 17826360 10.1016/j.modgep.2007.07.004 PMC2080859

[B64] Serafini T Colamarino SA Leonardo ED Wang H Beddington R Skarnes WC Tessier-Lavigne M Netrin-1 is required for commissural axon guidance in the developing vertebrate nervous system Cell 1996 87 1001 1014 8978605 10.1016/s0092-8674(00)81795-x

[B65] Grieshammer U Le M Plump AS Wang F Tessier-Lavigne M Martin GR SLIT2-mediated ROBO2 signaling restricts kidney induction to a single site Dev Cell 2004 6 709 717 15130495 10.1016/s1534-5807(04)00108-x

[B66] Nural HF Mastick GS Pax6 guides a relay of pioneer longitudinal axons in the embryonic mouse forebrain J Comp Neurol 2004 479 399 409 15514979 10.1002/cne.20317 PMC2080865

[B67] Rice WR Gaines SD Extending nondirectional heterogeneity tests to evaluate simply ordered alternative hypotheses Proc Natl Acad Sci U S A 1994 91 225 226 8278369 10.1073/pnas.91.1.225 PMC42919

[B68] Lin L Rao Y Isacson O Netrin-1 and slit-2 regulate and direct neurite growth of ventral midbrain dopaminergic neurons Mol Cell Neurosci 2005 28 547 555 15737744 10.1016/j.mcn.2004.11.009

