# Supplementary material for: Pioneer midbrain longitudinal axons navigate using a balance of Netrin attraction and Slit repulsion
Source: Neural Dev. 2014 Jul 24;9:17. doi: 10.1186/1749-8104-9-17 (PMC4118263; doi:10.1186/1749-8104-9-17)
Supplement: Additional file 1 — Quantification of MLF distance from the midline. Example showing the measurements made to compare the distance of MLF axons from the midline. Differences in developmental stage were normalized relative to the width (large bracket, wr1r2) of neural tissue at the r1/r2 border. [file 1749-8104-9-17-S1.pdf]

Additional file 1: Quantification of MLF distance from the midline

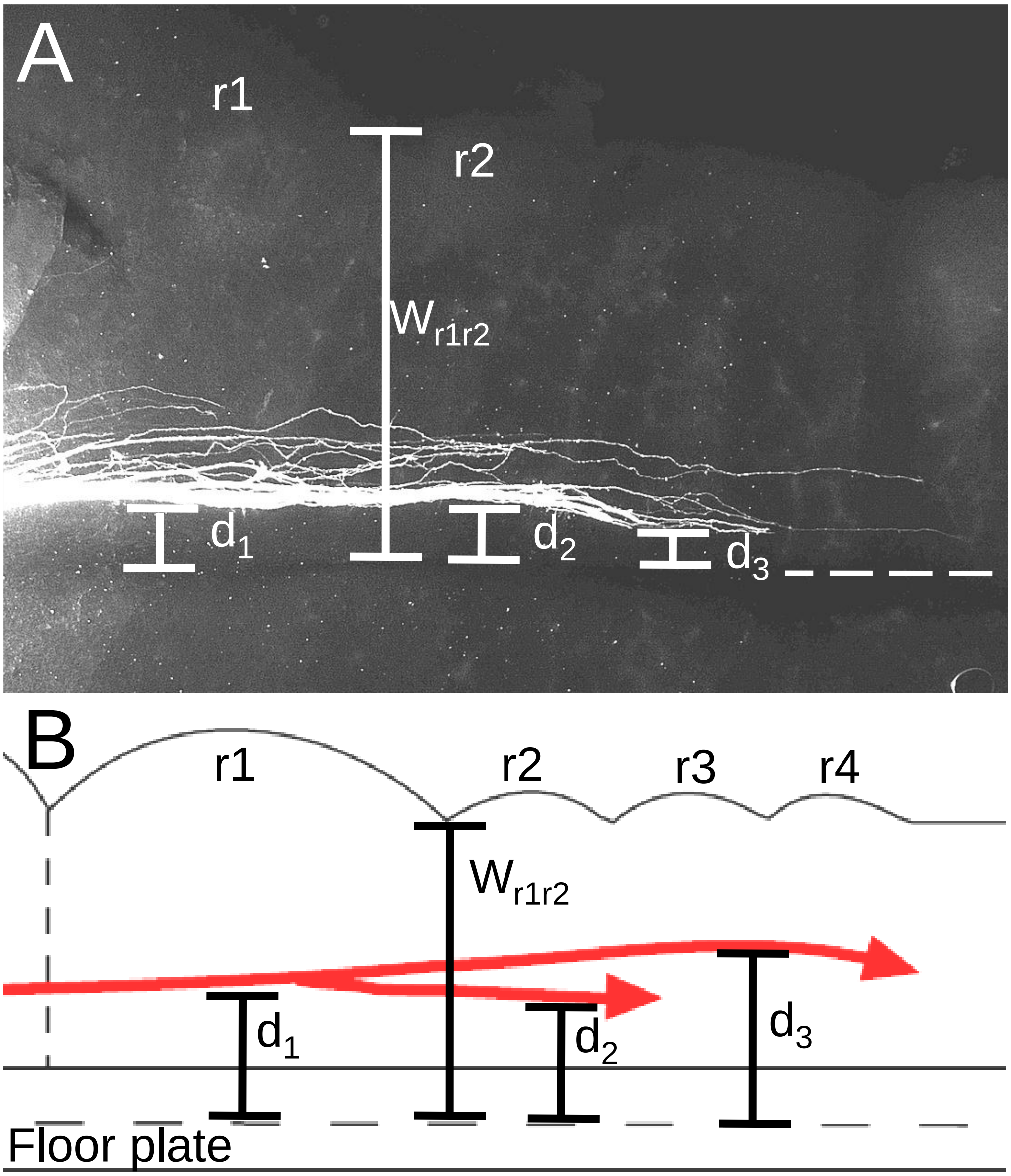

C

$$\text{Normalized Average Distance} = \frac{\frac{d_1}{w_{r1r2}} + \frac{d_2}{w_{r1r2}} + \frac{d_3}{w_{r1r2}} + \cdots \frac{d_n}{w_{r1r2}}}{n}$$
