# Supplementary material for: Pioneer midbrain longitudinal axons navigate using a balance of Netrin attraction and Slit repulsion
Source: Neural Dev. 2014 Jul 24;9:17. doi: 10.1186/1749-8104-9-17 (PMC4118263; doi:10.1186/1749-8104-9-17)

Additional file 2: Netrin1 mutant longitudinal axons retain wildtype axon numbers and DCC expression.

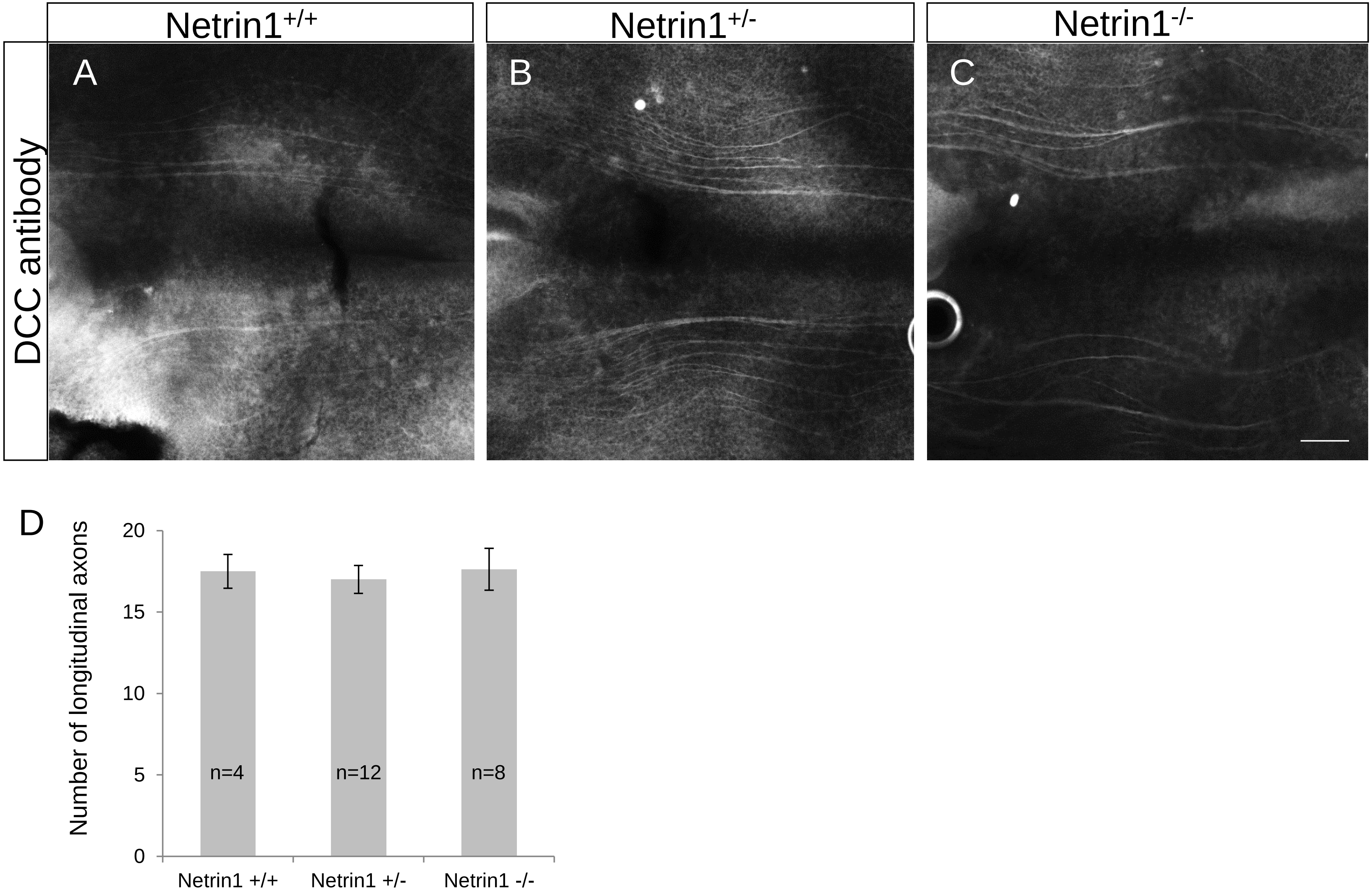

Supplement: Additional file 2 — Netrin1 mutant longitudinal axons retain wild-type axon numbers and DCC expression. (A-C) Whole mount embryos on E9.5 labeled with DCC antibody. The DCC labeling intensity was consistently lower in Netrin1+/+ control embryos. (D) Quantification of numbers of longitudinal axons on E9.5, by counting longitudinal axons on each side of the hindbrain in images of whole mounts. The n numbers indicate the number of embryos analyzed for each genotype; the error bars indicate SEM. By ANOVA analysis, there were no significant differences in axon number between the genotypes. Both ventral and dorsal axons were included in these counts. The size of the most ventral bundle, consisting of the MLF axons, was similar between genotypes, but tight fascicles made it difficult to determine the number of individual axons. Scale bar: 100 μm. [file 1749-8104-9-17-S2.pdf]
