# Supplementary figures and images for: Pioneer midbrain longitudinal axons navigate using a balance of Netrin attraction and Slit repulsion
Source: Neural Dev. 2014 Jul 24;9:17. doi: 10.1186/1749-8104-9-17 (PMC4118263; doi:10.1186/1749-8104-9-17)

**Additional file 3. Netrin1 mutants retain normal hindbrain floor plate size and specification.**

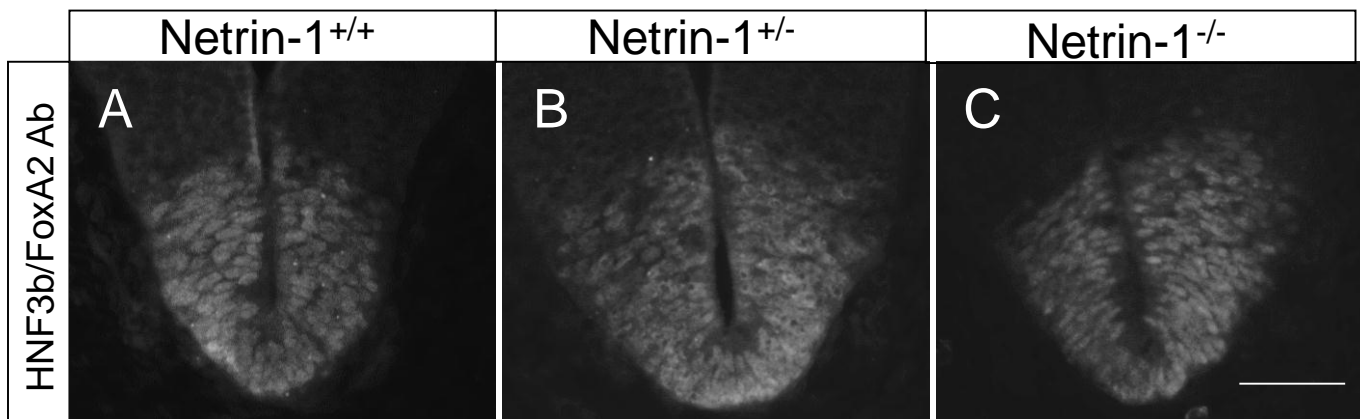

Supplement: Additional file 3 — Netrin1 mutants retain normal hindbrain floor plate size and specification. Sections through E9.5 hindbrain labeled with the 4C7antibody against HNF3b/FoxA2, a transcription factor expressed in a domain including the floor plate and adjacent ventral cells. The morphology and size of the floor plate domain appears similar in controls and Netrin1 mutants. Scale bar: 100 μm. [file 1749-8104-9-17-S3.pdf]

Additional file 4. Genotyping of Netrin1 mutant embryos.

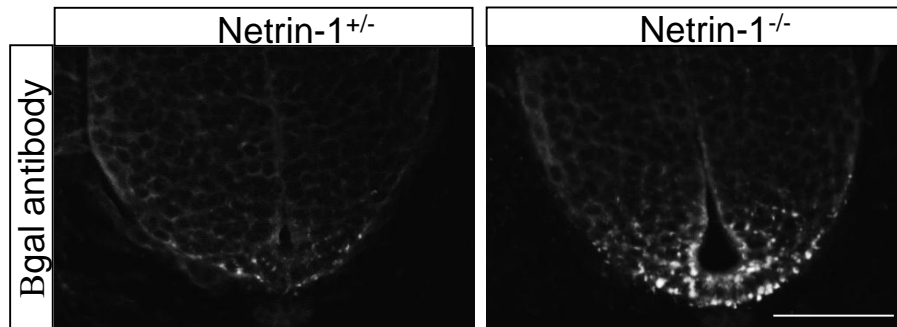

Supplement: Additional file 4 — Genotyping of Netrin1 mutant embryos. Beta-galactosidase antibody labeling of spinal cord sections. Note the higher intensity of antibody labeling in homozygous mutants. Scale bar: 100 μm. [file 1749-8104-9-17-S4.pdf]
